# Relationships between Instrumental and Sensory Quality Indices of Shine Muscat Grapes with Different Harvesting Times

**DOI:** 10.3390/foods11162482

**Published:** 2022-08-17

**Authors:** Kyeong-Ok Choi, Youn Young Hur, Seo Jun Park, Dong Hoon Lee, Su Jin Kim, Dongjun Im

**Affiliations:** Fruit Research Division, National Institute of Horticultural and Herbal Science, Wanju 55365, Korea

**Keywords:** Shine Muscat grapes, ripening, instrumental quality indices, sensory properties

## Abstract

The effects of instrumental quality indices on the sensory properties of Shine Muscat grapes harvested 16, 18, 20, and 22 weeks after full bloom (WAFB) were investigated. The berries harvested at 20 and 22 WAFB gained higher sweetness scores than those harvested at 16 and 18 WAFB, showing similar trends to that of total soluble solids (TSS) content. The sourness, astringency, and firmness scores were not significantly different among the samples. The flavor score showed a trend similar to that of sweetness perception. The higher flavor score in the berries harvested at 20 and 22 WAFB seemed to be derived from the development of floral aroma compounds, including linalool and its derivatives, with ripening. Consumer acceptance was highly correlated with sweetness and flavor perceptions. It was concluded that the TSS content and development of floral aroma compounds are the key quality parameters for Shine Muscat grapes, satisfying consumer acceptability in the market.

## 1. Introduction

Shine Muscat, a cross between Akitsu−21 (a hybrid of ‘Steuben’ (*V. labruscana*) × ‘Muscat of Alexandria’ (*V. vinifera*)) and ‘Hakunan’ (*V. vinifera*), is the most popular table grape in Asian countries because of its strong muscat flavor, excellent sweetness edible-peel, and low sourness [1]. The popularity of Shine Muscat grape has led many grape growers to harvest and market immature berries, consequently decreasing the quality of Shine Muscat grape and thus consumer demand. In particular, low sweetness and muscat flavor are major issues.

To date, many studies on the quality assessment of grapes have been carried out [1,2,3,4,5,6]. Traditionally, grape quality and maturity have been assessed primarily by the total soluble solids (TSS) content and titratable acidity (TA). Hence, the Codex Alimentarius Commission adopted a minimum sweetness requirement of 16 °Bx [7]. Additionally, various alternative quality indices and conventional and novel analysis techniques, such as TSS/TA, berry size and weight, color, flavor intensity, and texture analysis, have been used to evaluate grape quality and maturity [1,2]. Jayasena and Cameron [2] reported that for Crimson Seedless grapes, TSS/TA was better at reflecting consumer acceptability than TSS and TA alone. Pothen and Nuske [3] developed an automated assessment and mapping technique using image-based color analysis to evaluate and predict the quality and harvesting time of grapes. Melo et al. [4] evaluated the effects of berry size of wine grapes on wine quality and found that the extractability of anthocyanins and phenolic compounds that affect the final wine quality was higher in small berries than in medium and large berries. Sasaki et al. [5] evaluated and compared the volatile aroma compounds of Ruby Roman grapes with different grape varieties. They reported that 15 esters not found in the other grape varieties were the aroma compounds representing the fruity flavor of the Ruby Roman grape variety. Giacosa et al. [6] applied a combination of three different mechanical tests (texture profile analysis, cutting, and dentures) to evaluate the perceived firmness and crunchiness of different grape varieties. Vallone et al. [8] applied a non-destructive near-infrared spectroscopic technique for the quality evaluation of wine grapes for mechanical harvesting. However, these instrumental measures sometimes make it difficult to predict the sensory characteristics of the test objects.

Sensory analysis is considered a major approach for evaluating the organoleptic qualities of natural and processed foods. The perception of foods is a complex process and a combination of taste, visual, olfactory, auditory, and texture recognized by the human senses. Basile et al. [9] investigated the correlation between NIR spectroscopy data and the sensory properties of table grapes to understand consumer preferences. Ha et al. [10] evaluated the relationship between instrumental quality indices and consumer sensory acceptance of refrigerated-stored Campbell Early and Kyoho grapes. Sasaki et al. [5] evaluated the quality of Ruby Roman grapes using instrumental measures and sensory evaluations. Nevertheless, to our knowledge, studies evaluating the relationships between instrumental quality indices and sensory characteristics of Shine Muscat grapes have rarely been conducted. In the present study, the influence of instrumental quality indices on the sensory attributes of Shine Muscat grapes was evaluated to improve the understanding of consumer preferences.

## 2. Materials and Methods

### 2.1. Study Site, Experimental Design and Treatments

Bovine serum albumin (BSA; Fraction V, 96–98%), tannic acid, triethanolamine, and acetonitrile were purchased from Sigma-Aldrich (St. Louis, MO, USA). Urea was purchased from Promega (Madison, WI, USA). A sodium hydroxide standard solution (0.1 M) was purchased from Yakuri Pure Chemicals (Kyoto, Japan). All the analytical aroma standards were purchased from Sigma-Aldrich. All other reagents used were of analytical grade. Shine Muscat grapes were cultivated in vineyards in Ansung, Korea, in 2020. The experiment was a randomized block design with three treatments and three replicates. Each block consisted of five vines that were spaced at a fixed distance of 5 × 2.7 m. The grape berries were treated with a combination of gibberellic acid and thidiazuron (25 and 2 ppm, respectively) twice, at 2 and 14 days after full bloom. Berry thinning was performed by removing approximately half the berries. The grapes were harvested at 6, 8, 10, 12, 14, 16, 18, 20, and 22 weeks after full bloom (WAFB), from July to October 2020. The harvested grape samples were grouped according to the WAFB. Grape berries from twenty clusters collected from different vines were analyzed immediately after harvest. Ten clusters were subjected to instrumental analyses and the other ten clusters were used for sensory analysis.

### 2.2. Total Soluble Solids Content and Titratable Acidity

Ten berries were randomly selected from each group and free-flowing juice was collected. The TSS content of the juice was determined using a digital refractometer (PR-32 Alpha; ATAGO Co. Ltd., Tokyo, Japan) and expressed as °Bx TA was determined using an automatic titrator (TitroLine Easy; SI Analytics GmbH, Mainz, Germany). Titration was performed on 5 mL juice diluted in 20 mL distilled water using a 0.1 M NaOH standard solution until the sample solution reached a pH of 8.2. TA was expressed as tartaric acid equivalents. The Brix/acid ratio was calculated by dividing the °Bx value by the TA of grape juice samples.

### 2.3. Polymeric Tannin Content

The polymeric tannin content (PTC) in the extracts was determined using a BSA precipitation assay [11] with slight modifications. Grape skins were manually separated from the pulp using a flat spatula and immediately soaked in deionized water. The collected skins were lyophilized for 48 h after washing, and the dried skins were pulverized using an electric grinder. Ground skin samples were stored in a desiccator protected from light prior to use. For the extraction of phenolic compounds, 0.3 g of powder was added to 10 mL of an extraction solvent consisting of water, acetone, and methanol (0.36:0.48:0.16, *v*/*v*/*v*). The mixture was thoroughly vortexed, and extraction was performed twice in an ultrasonic bath (DH.WUC.D10H, DAIHAN Scientific, Wonju, Korea) at 20 °C for 1 h (30 min each). The extract was filtered and stored at 4 °C prior to analysis. In a 2 mL microtube, 0.2 mL of the extract was mixed with 1 mL of BSA solution (1 mg/mL BSA in a washing buffer). The mixture was vortexed thoroughly, incubated for 10 min, and centrifuged at 10,000 rpm for 2 min. The supernatant was removed, and the precipitate was washed with 1 mL of washing buffer (170 mM NaCl in 200 mM acetic acid, pH 4.9) to remove free acids and monomeric and dimeric tannins. Subsequently, 875 µL of 8.3 M aqueous urea solution with 5% triethanolamine (pH 7.0) was added, vortexed, and incubated for 10 min at room temperature (25 ± 2 °C) to isolate polymeric tannin from the protein/tannin complex. For color development, 175 µL of resuspended tannin solution was mixed with 25 µL of FeCl_3_ solution (10 mM FeCl_3_ in 10 mM HCl) using a 96-well microplate. After incubation for 10 min in a microplate reader with shaking for the first 2 min, the absorbance of the reaction mixture was measured at a wavelength of 510 nm against the blank solution. PTC was calculated using a tannic acid (TA) calibration curve. Different concentrations of TA in the urea solution (10, 50, 100, 200, and 400 µg/mL) were used to prepare the calibration curve. PTC was expressed as milligram tannic acid equivalent/g dry weight of skin powder (mg TAE/g DW).

### 2.4. Texture Analysis

Thirty berries with regular shapes and sizes were randomly selected from the grape bunches, and their textural properties were measured using a texture analyzer (TA1, AMETEK, Berwyn, PA, USA). The tests were performed on the day after harvest. A penetration test was performed using a 5 mm cylindrical stainless probe connected to a 45 N loading cell under the following conditions: 100 mm/min test speed, 1.0 N trigger force, and 80% strain. From the force-distance curve, the maximum force and the average value of the forces measured after skin rupture were calculated to obtain the skin rupture strength and flesh firmness, respectively, using Nexygen Plus software version 3.0. Grape berries were analyzed at room temperature (25 ± 2 °C).

### 2.5. Volatile Free Aroma Compounds

#### 2.5.1. Free Aroma Extraction and Quantification

The extraction and quantification of free aroma compounds were performed using a headspace solid-phase microextraction coupled with gas chromatography–mass spectrometry (HS-SPME/GC-MS). Grape berries (100 g) were randomly selected and ground using an electric grinder. Grape juice was separated from the pomace by centrifugation and filtration. Extraction and GC/MS analyses of the volatile aroma compounds were performed under pre-established conditions. For the extraction of volatile aroma compounds, 10 mL grape juice was added to a 20 mL volume headspace vial containing 3 g NaCl and acetonitrile as an internal standard, and the sample vial was incubated at 50 °C with constant stirring for 1 h. After incubation, an SPME fiber (50/30 µm DVB/CAR/PDMS; Supelco, Bellefonte, PA, USA) was introduced into the headspace of the sample vial for 20 min to adsorb the volatile aroma compounds. The SPME fiber was directly injected into the injection port of a gas chromatograph (6890N, Agilent Technologies, Santa Clara, CA, USA) equipped with an Agilent 5975 Series Mass Selective Detector. An HP-INNOWax capillary column (30 mm × 0.32 mm × 0.25 µm; Agilent Technologies) was used to separate the volatile aroma compounds, and purified helium gas was used as a carrier gas at a constant flow rate of 2 mL/min. The injection port temperature was set to 250 °C, and the purge flow to the split vent was set to 144.9 mL/min for 1 min. The oven temperature was initially held at 40 °C for 5 min, then increased to 250 °C at a rate of 5 °C/min, and finally held at 250 °C for 5 min. The mass detector was operated in the positive-ion electron-impact ionization mode at 70 eV with a scan range of 50–700 m/z. Volatile aroma compounds were identified using their mass spectra from the NIST 11 (National Institute of Standards and Technology) mass spectral library and Kovat retention indices. The concentrations of the volatile aroma compounds were estimated using the following Equations [12]:

(1) $$RF_{SC}=\frac{A_{IS}\times C_{SSC}}{C_{IS}\times A_{SC}}$$

(2) $$C_{SC}=\frac{C_{IS}\times A_{SC}\times RF_{SC}}{A_{IS}}$$

where *RF_SC_* is the response factor for a specific compound, *A_IS_* is the peak area of the internal standard, *C_SSC_* is the concentration of the authentic standard of a specific compound, *C_IS_* is the concentration of the internal standard, *A_SC_* is the peak area of a specific compound, and *C_SC_* is the concentration of a specific compound.

#### 2.5.2. Odor Activity Value

Odor activity value (OAV) analysis was performed by dividing the concentration of each aroma compound by its perception threshold in water reported in the literature [13,14,15,16,17,18]. OAV values higher than 1 were applied to a principal component analysis using OriginLab Pro software (OriginLab Corporation, Northampton, MA, USA) to classify the Shine Muscat grapes of different ripening degrees according to the active odorants.

### 2.6. Sensory Evaluation

The sensory properties of Shine Muscat grapes at different ripening degrees were evaluated with the approval of the Institutional Review Board (IRB) in May 2021 (P01-202105-13-004). A panel of 33, 26 women and 7 men, aged 20–60 years who had no repulsion against muscat flavor, were recruited from the National Institute of Horticultural and Herbal Science. A commercial grape at 21 °Bx, purchased from a local market, was used as a representative grape sample and for training the panelists. The tests were performed on four grape samples harvested at 16, 18, 20, and 22 WAFB with TSS values higher than 16 °Bx as specified by the Codex Standard 255-2007 for table grapes [7]. Each test was performed the day after harvest. All grape samples were stored at 4 °C prior and allowed to equilibrate to room temperature before testing. The sensory parameters were sweetness, sourness, firmness, flavor, and overall acceptance. For the test, 30 g of grape berry was served to the panelists, and water was provided freely to clean their palates. The intensities of all sensory parameters and the overall acceptance of the tested grape samples were scored on a five-point scale.

### 2.7. Statistical Analysis

All experiments were performed with at least two biological and three technical replicates, and the results were statistically analyzed using the SPSS software (IBM, Armonk, NY, USA). Analysis of variance (ANOVA) was applied, followed by Tukey’s multiple comparison test for mean comparisons. Differences were considered statistically significant at *p* < 0.05. The relationships among the sensory attributes were determined by a Pearson’s correlation coefficient test using OriginLab Pro software (OriginLab Corporation, Northampton, MA, USA) and are depicted in a heatmap.

## 3. Results and Discussion

### 3.1. Total Soluble Solids Content and Titratable Acidity

**Figure 1:**
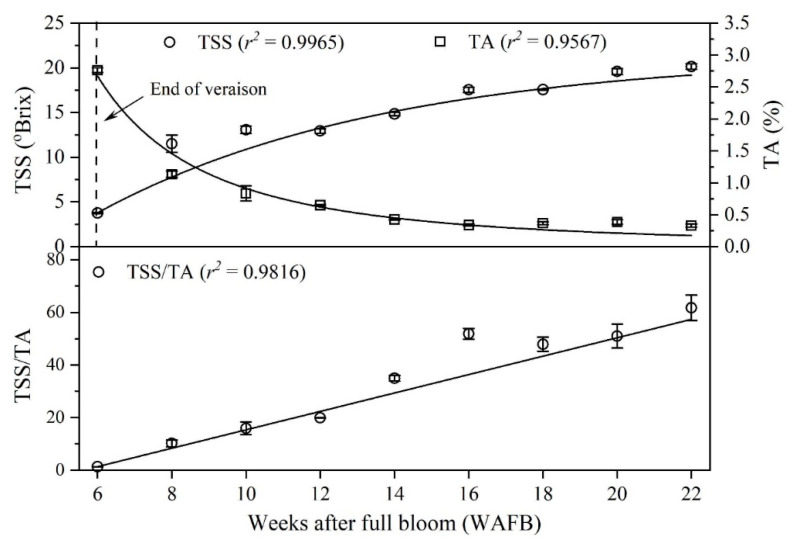
Changes in total soluble solids (TSS) content, titratable acidity (TA), and Brix/acid ratio of Shine Muscat grapes during ripening.

The onset of berry ripening was determined based on TSS and TA. Dramatic changes in the physical, chemical, and physiological properties of grape berries can be observed at veraison. An increase in berry size and volume, accumulation of sugars, tannins, and anthocyanins, and degradation of organic acids and chlorophylls are commonly observed after veraison [19]. Monitoring the changes in TSS and TA in grape berries is an important indicator for judging the end of veraison, representing the onset of ripening [20], especially for white grape cultivars. TSS showed a rapid increase between 6 and 8 WAFB due to the accumulation of sugars and then gently increased during the ripening period, while the change in TA showed the opposite trend. The TSS of the berry extract was 3.8 °Bx at 6 WAFB, increased to 11.5 °Bx at 8 WAFB, and then reached a maximum of 20.1 °Bx at 22 WAFB (Figure 1 and Table 1). On the other hand, the TA was 2.8% at 6 WAFB, decreased to 1.1%, reached approximately 0.3% at 16 WAFB, and remained constant thereafter (Figure 1 and Table 1). The rapid increase in TSS and reduction in TA starting at 6 WAFB visibly indicates the onset of grape berry ripening, as shown in Figure 1.

The Shine Muscat grape berry surpassed the minimum quality requirement of 16 °Bx specified by the Codex Standard 255-2007 for table grapes [7] at 16 WAFB as 17.5 °Bx (Table 1). The Brix/acid ratio of grape berries was very low (1.4) at the beginning of ripening because of the low sugar and high acid content and then reached 20 at 12 WAFB, which is the minimum quality requirement specified by the Codex Standard 255-2007 for table grapes [7]. The Brix/acid ratio rapidly increased with ripening by 16 WAFB due to the accumulation of sugars and degradation of organic acids and plateaued thereafter. The Brix/acid ratio is considered an alternative indicator of °Bx for determining the ripeness of grapes [21] because of the variation in both sugar and acid levels in grape berries from year to year under the same cultivation conditions. Furthermore, the Brix/acid ratio is more important with respect to consumer acceptance than either °Bx or acid concentration alone [22]. Jayasena [2] investigated the effects of the Brix/acid ratio on consumer acceptance and found that consumer acceptance dramatically increased as the ratio of Brix to acid value increased from 20 to 40.

### 3.2. Polymeric Tannin Content and Textural Properties

Changes in PTC and textural properties of Shine Muscat grapes during ripening were investigated, and the results are presented in Table 1. The PTC of grape berries was significantly lower in the berries harvested at 16 WAFB than in the others. The polymeric tannin content is an important parameter for judging the eating and drinking quality of grapes and wines since polymeric tannin, rather than other phenolic constituents, such as anthocyanins and proanthocyanidins is responsible for the astringent sensation [11]. In contrast, the skin rupture strength and flesh firmness values of Shine Muscat grapes were significantly higher in berries harvested at 16 WAFB than in the others. The textural characteristics of grapes are influenced by berry size and density [23,24] rather than the degree of ripeness. Our results cannot explain the parameters affecting the texture properties of grape berries; however, they can be used to evaluate the relationship between the instrumental and sensory texture properties of grapes.

### 3.3. Free Volatile Aroma Compounds 

The changes in volatile free aroma compounds in the juice of Shine Muscat grapes from 16 to 22 WAFB during ripening are presented in Table 2. The total alcohol content tended to decrease by 20 WAFB and then increased thereafter. Six-carbon alcohols (C6-alcohols) such as (*E*)-2-hexen-1-ol, (*Z*)-3-hexen-1-ol, and 1-hexanol comprised the majority (>98%) of the total alcohol content. In particular, (*E*)-2-hexen-1-ol, and 1-hexanol significantly affected total alcohol content. These alcohol species are responsible for the herbaceous flavor of unripe grapes [25]. The total aldehyde content tended to increase with ripening. The major species were hexanal and (*E*)-2-hexenal, which accounted for >99% of the total aldehyde content. The result was in agreement with that of a previous study [26]. Aldehydes partly contribute to the herbaceous characteristics of grapes. Monoterpenes are the major volatile compounds responsible for the intensity of muscat flavor in Shine Muscat grapes. Among them, linalool, geraniol, nerol, citronellol, and α-terpineol are the major constituents of the floral and fruity flavors of Muscat grapes [19]. However, the concentrations of nerol, citronellol, and α-terpineol were relatively lower than those of geraniol and linalool. The concentration of geraniol tended to decrease with ripening, which is in agreement with a previous study by Wilson et al. [27]. They reported that the geraniol concentration in Muscat grapes was higher than that of other monoterpene compounds before veraison and then decreased during ripening. The concentration of linalool, the most abundant monoterpene compound in ripe berries, tended to constantly increase during ripening. Thus, the total monoterpene content was dependent on linalool content. Oxidized forms of linalool, including furanoid and pyranoid forms, were also present, and their concentrations tended to increase during ripening.

**Figure 2:**
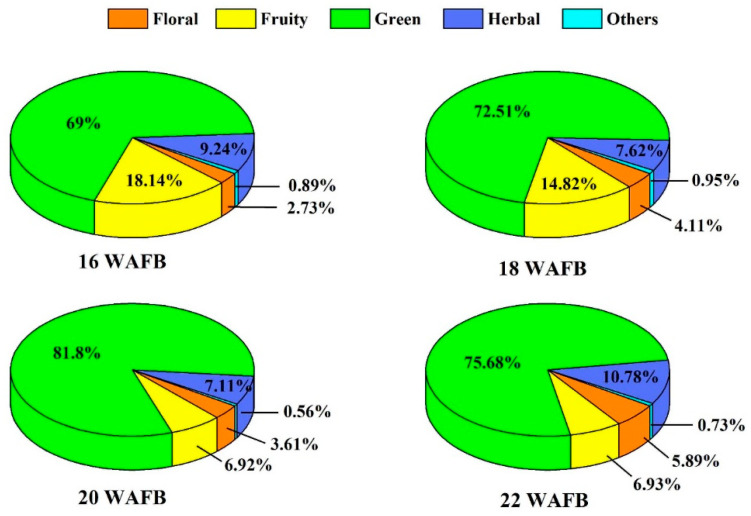
Pie charts representing the percentages of free volatile aroma compounds classified into odor types in Shine Muscat grapes harvested at 16, 18, 20, and 22 weeks after full bloom (WAFB): Others include citrus, earthy, fatty, minty, tropical, waxy, and aldehydic odors.

The volatile aroma compounds were grouped according to their aroma types, and their proportions are shown in Figure 2. Green aroma compounds occupied the highest proportion (69–82%), regardless of the ripening degree, which seemed to be largely affected by (*E*)-2-hexenal and hexanal. It was presumed that green and herbaceous flavors would govern the overall flavor. The proportion of fruity aroma compounds tended to decrease owing to the decreasing trend of (*E*)-2-hexen-1-ol with increasing ripening. Meanwhile, the proportion of floral aroma compounds tended to increase because of the increasing linalool content. The proportion of herbal aroma compounds was significantly affected by the concentration of 1-hexanol.

### 3.4. Active Odorants

The odor thresholds of the aroma compounds detected in Shine Muscat grapes and their odor activity values are presented in Table 3. A total of 44 volatile aroma compounds (10 alcohols, 6 aldehydes, 1 ester, 5 ketones, and 22 monoterpenes) were detected in Shine Muscat grape berries using GC/MS analysis. Among them, only eight compounds, (*E*)-2-hexen-1-ol, 1-hexanol, (*Z*)-3-hexenal, (*E*)-2-hexenal, hexanal, (*E*)-linalool oxide, (*E*)-β-damascenone, and linalool, were found to be active odorants (OAV > 1). Thus, these eight volatile compounds seemed to be key aroma compounds responsible for the overall aroma characteristics of grape berries. However, the other compounds with OAV less than 1 and glycosidic aroma compounds could affect the aroma characteristics. For example, aroma glycosides undergo hydrolysis by β-glucosidase in the mouth, enhancing the fruit flavor [28].

The OAV of (*E*)-2-hexen-1-ol, the only active compound representing the fruity scent, tended to decrease with ripening, while 1-hexanol with an herbal scent was highest in berries harvested at 22 WAFB. The OAVs of aldehyde compounds, including (*Z*)-3-hexenal, (*E*)-2-hexenal, and hexanal, responsible for green and herbaceous scents, were relatively higher than those of other volatile compounds. The OAVs of linalool and its derivative (linalool oxide furanoid), representing a floral scent, tended to increase with ripening. (*E*)-β-Damascenone, a floral volatile, is an important active odorant primarily contributing to the flavor of Shine Muscat grape despite its presence in very low to trace amounts due to its very low odor threshold (0.002 ug/L in water). This result is in agreement with that of a previous report [18].

**Figure 3:**
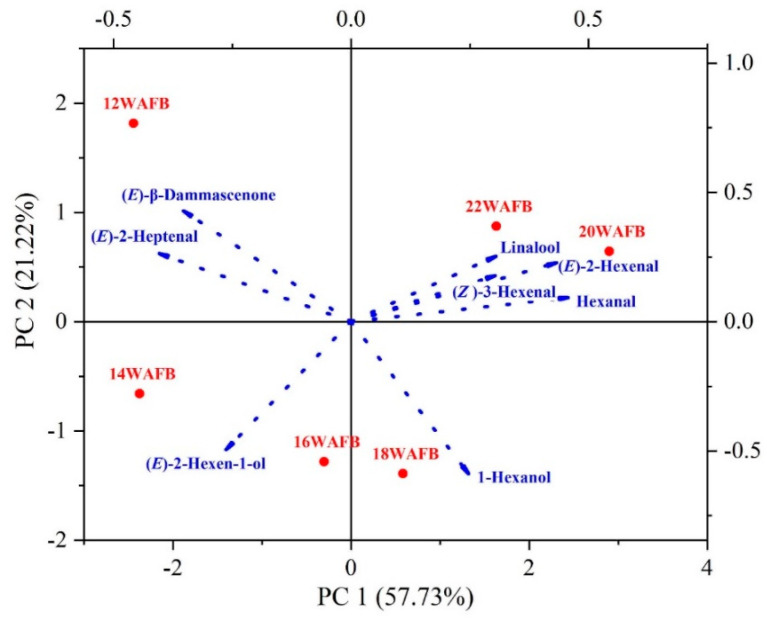
Biplot of principal component analysis (PCA) for active odorants in Shine Muscat grape during ripening from 12 to 22 weeks after full bloom (WAFB): Red dots represent grape samples harvested at different periods.

Principal component analysis (PCA) was performed with eight active odorants, and the results are shown in Figure 3. The two principal components (PC1 and PC2) accounted for 79% of total variance (57.73% and 21.22%, respectively). The first component, PC1, was composed of monoterpenes and aldehydes such as linalool, hexanal, (*E*)-2-hexenal, and (*Z*)-3-hexenal, while, the second component, PC2, was composed primarily of (*E*)-β-damascenone. The grape harvested at 12 WAFB was located on the upper-left quadrant plane, which was positively correlated with (*E*)-β-damascenone. The grapes harvested at 14 and 16 WAFB were located in the lower-left quadrant, which was positively correlated with (*E*)-2-hexen-1-ol; those harvested at 18 WAFB were positively correlated with 1-hexenol; and those harvested at 20 and 22 WAFB were grouped in the upper-right quadrant, which represents a positive correlation with hexanal, (*E*)-2-hexenal, (*Z*)-3-hexenal, and linalool. The flavor of grapes harvested at 12, 14, and 16 WAFB seemed to be characterized by fruity, green, and herbaceous flavor owing to the dominant influence (*E*)-β-damascenone and (*E*)-hexen-1-ol, while the flavor of grapes harvested at 18 WAFB seemed to be primarily affected by 1-hexanol and also linalool, giving rise to a floral flavor. The grapes harvested at 20 and 22 WAFB were clearly distinguished from the others, being largely affected by the odor activity of linalool. Thus, it was clearly observed that the primary aroma compounds of Shine Muscat grapes changed from C13-norisoprenoids, C6-alcohols, C6-aldehydes, and finally to monoterpenes, with ripening.

### 3.5. Sensory Property

**Figure 4:**
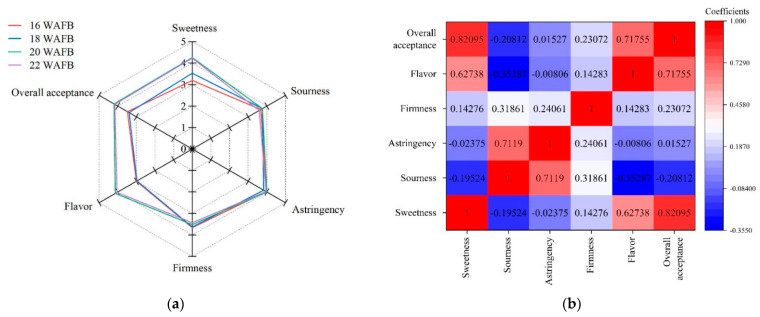
Schematic representations of (**a**) a radar plot of hedonic sensory properties of Shine Muscat grapes harvested at 16, 18, 20, and 22 weeks after full bloom (WAFB), and (**b**) heatmap of Pearson’s correlation coefficient matrix between sensory attributes.

Sensory evaluation was performed on Shine Muscat grapes harvested at 16, 18, 20, and 22 WAFB; the results are shown in Table 4 and Figure 4. The hedonic sensory scores for each sensory attribute are presented in the radar plot in Figure 4a. The sweetness scores showed a similar pattern to the instrumental total soluble solid contents (TSS) and TSS/TA of grape samples, showing an increasing trend of °Bx and TSS/TA value as ripening progressed (Figure 1). Thus, the grape samples harvested at 20 and 22 WAFB had significantly higher sweetness scores than those harvested at 16 and 18 WAFB (Table 4). The panelists could distinguish the perception of sweetness at a 2 °Bx difference. Flavor scores showed a trend similar to that of sweetness. As described in the active odorant analysis (Table 3), the odor activities of the key aroma compounds changed during ripening. From the results of sensory flavor attributes and active odorant analysis, it was assumed that the panelists captured fruity, green, and herbaceous flavors from the grapes harvested at 16 and 18 WAFB, owing to the dominant influence of (*E*)-2-hexenal, hexanal, (*E*)-hexen-1-ol, and 1-hexanol. For the grapes harvested at 20 and 22 WAFB, linalool and its derivative seemed to develop the floral flavor significantly. An appreciable difference in the flavor scores between grapes obtained after 20 and 22 WAFB and those obtained after 16 and 18 WAFB strongly supports this result (Table 2). However, there were no significant differences in sourness scores among the samples, which is consistent with the trend of instrumental TA values. Although the instrumental measures of PTC and firmness of the berries harvested at 16 WAFB were significantly higher than those of the others, the sensory astringency and firmness scores showed no significant differences among the samples. Small differences in the instrumental measures of tannin content and mechanical texture properties of ripened Shine Muscat grape berries rarely affect their sensory astringent sensation and firmness. Regarding consumer acceptance, the correlation coefficients presented in Figure 4b indicate that sweetness and flavor were highly positively correlated with overall acceptability (*p* < 0.01). In contrast, the sourness was negatively correlated with the overall acceptability (*p* < 0.05). Astringency and firmness were not significantly correlated (*p* > 0.05). It was obvious that the sweetness and flavor attributes strongly affected the overall acceptability compared with the other attributes.

## 4. Conclusions

The relationships between instrumental quality indices and sensory properties of Shine Muscat grapes harvested 16, 18, 20, and 22 weeks after full bloom (WAFB) were evaluated. The sweetness scores showed similar trends to the total soluble solids (TSS) content of berries. However, the sourness, astringency, and firmness scores showed no significant differences among the samples. The flavor score showed a trend similar to that of sweetness perception. The higher flavor score in the berries harvested at 20 and 22 WAFB seemed to result from the development of floral monoterpene compounds, in particular linalool and its derivatives, with ripening. Principal component analysis indicated that the sensory flavor characteristics of Shine Muscat grapes harvested from 16 to 22 WAFB changed from fruity and herbaceous to floral with the alteration of key active aroma compounds with ripening. From the results, it was concluded that sweetness and flavor are the most important quality indices affecting consumer preference for Shine Muscat grapes. The instrumental measurement of sugar content and aroma compounds could provide an excellent quality assessment of ripened Shine Muscat grapes.

## Figures and Tables

**Table 1:** Changes in total soluble solids content, titratable acidity, polymeric tannin content, and textural properties of Shine Muscat grapes during ripening from 16 to 22 WAFB.

^1^ WAFB	^2^ TSS (°Bx)	^3^ TA (%)	^4^ PTC (mgTAE/gDW)	Textural Properties	
				Skin Rupture Strength (N)	Flesh Firmness (N)
16	17.5 ± 0.3 ^b^	0.34 ± 0.01 ^a^	9.1 ± 0.3 ^b^	10.99 ± 1.26 ^a^	2.48 ± 0.32 ^a^
18	17.6 ± 0.1 ^b^	0.37 ± 0.02 ^a^	10.2 ± 0.5 ^a^	6.8 ± 1.11 ^b^	1.73 ± 0.14 ^b^
20	19.6 ± 0.3 ^a^	0.39 ± 0.04 ^a^	10.0 ± 0.4 ^a^	5.99 ± 0.83 ^b^	1.82 ± 0.25 ^b^
22	20.1 ± 0.3 ^a^	0.33 ± 0.02 ^a^	10.1 ± 0.4 ^a^	6.14 ± 0.39 ^b^	1.99 ± 0.12 ^b^

^1^ WAFB: Weeks after full bloom.; ^2^ TSS: Total soluble solid content; ^3^ TA: Titratable acidity; ^4^ PTC: Polymeric tannin content expressed in tannic acid equivalent. Different letters (a and b) within the same column indicate significant differences at *p* < 0.05.

**Table 2:** Changes in composition and concentration of free volatile aroma compounds in Shine Muscat grapes during ripening.

Group	Aroma Compounds	^1^ RI	Weeks After Full Bloom					
			12	14	16	18	20	22
Alcohol	2-Ethyl-1-hexanol	1510	4.0(0.3) ^c^	4.4(0.2) ^bc^	4.7(0.3) ^b^	5.4(0.1) ^a^	5.4(0.3) ^a^	4.6(0.2) ^bc^
	1-Heptanol	1472	0.3(0.03) ^b^	0.5(0.1) ^a^	0.4(0.1) ^ab^	-	-	-
	1-Hexanol	1373	168.6(65.2) ^d^	366.9(18.8) ^c^	724.3(18.4) ^b^	938.7(56.1) ^a^	653.8(98.6) ^b^	403.1(39.5) ^c^
	(*E*)-2-Hexen-1-ol	1426	294.7(79.6) ^cd^	893.6(41.0) ^a^	623.1(84.0) ^b^	439.3(22.8) ^c^	279.1(48.7) ^d^	279.5(19.9) ^d^
	(*E*)-3-Hexen-1-ol	1382	-	0.9(0.03) ^a^	-	0.3(0.02) ^b^	0.8(0.1) ^a^	0.5(0.1) ^b^
	(*Z*)-3-Hexen-1-ol	1400	33.1(1.4) ^c^	42.9(0.3) ^b^	54(2.5) ^a^	45.1(3.1) ^b^	29.1(0.6) ^c^	17.3(1.0) ^d^
	1-Nonanol	1679	0.2(0.01) ^a^	0.1(0.01) ^b^	0.1(0.01) ^b^	0.1(0.003) ^c^	0.1(0.002) ^c^	0.1(0.01) ^c^
	(*E*)-2-Hepten-1-ol	1529	-	0.1(0.01)	-	-	-	-
	1-Octanol	1577	0.5(0.03) ^a^	0.6(0.1) ^a^	-	-	0.3(0.08) ^b^	0.5(0.1) ^a^
	1-Octen-3-ol	1472	0.1(0.02) ^a^	0.2(0.04) ^a^	0.2(0.02) ^a^	0.2(0.04) ^a^	0.1(0.03) ^a^	0.1(0.03) ^a^
	(*E*)-2-Penten-1-ol	1335	-	0.5(0.04) ^c^	0.3(0.03) ^c^	0.4(0.03) ^c^	1.2(0.3) ^b^	2.2(0.3) ^a^
	Subtotal		501.5	1310.7	1407.1	1429.5	969.9	707.9
Aldehyde	Benzaldehyde	1525	2.9(0.1) ^b^	2.1(0.5) ^cd^	2.5(0.2) ^bc^	4.0(0.03) ^a^	1.8(0.2) ^d^	1.6(0.2) ^d^
	Hexanal	1101	773.6(79.2) ^c^	781.8(72.0) ^c^	1069.2(70.6) ^b^	1104.5(124.1) ^b^	1643.6(94.1) ^a^	1414.7(86.5) ^a^
	(*E*)-2-Heptenal	1329	0.8(0.1)	0.8(0.01)	-	-	-	-
	(*E*)-2-Hexenal	1215	1067(158.2) ^ab^	922.9(121.6) ^b^	1054.7(236.3) ^ab^	1127.1(182.3) ^ab^	1411.5(103.6) ^a^	710.2(55.5) ^b^
	(*Z*)-3-Hexenal	1154	4.3(0.4) ^e^	3.8(0.001) ^de^	4.6(0.2) ^cd^	5.2(0.4) ^c^	8.5(0.1) ^a^	6.6(0.3) ^b^
	Nonanal	1404	0.3(0.01) ^ab^	0.3(0.1) ^ab^	0.4(0.1) ^a^	0.3(0.1) ^ab^	0.2(0.01) ^bc^	0.1(0.004) ^c^
	(*E*)-2-Octenal	1435	-	-	-	0.2(0.02)	-	-
	Subtotal		1848.9	1711.7	2131.4	2241.3	3065.6	2133.2
Ester	Methyl salicylate	1761	0.7(0.1) ^b^	1.4(0.2) ^a^	0.4(0.1) ^c^	0.3(0.04) ^cd^	0.03(0.002) ^d^	0.03(0.01) ^d^
	Ethyl octanoate	1446	0.2(0.1)	-	-	-	-	-
	Subtotal		0.9	1.4	0.4	0.3	0.03	0.03
Ketone	(*E*)-β-Damascenone	1817	0.68(0.07) ^a^	0.21(0.01) ^b^	0.15(0.002) ^b^	0.02(0.003) ^c^	0.02(0.001) ^c^	0.04(0.004) ^c^
	2-Heptanone	1195	0.9(0.1) ^b^	1.2(0.1) ^a^	0.5(0.1) ^c^	0.2(0.1) ^d^	0.3(0.1) ^d^	0.2(0.04) ^d^
	sulcatone	1351	2.8(0.6) ^b^	3.6(1.0) ^ab^	3.3(0.8) ^ab^	3.9(0.4) ^ab^	4.6(0.5) ^a^	3.1(0.1) ^ab^
	2-Octanone	1296	4.1(0.5) ^a^	3.3(0.3) ^b^	1.7(0.003) ^c^	0.5(0.1) ^d^	0.4(0.1) ^d^	0.4(0.04) ^d^
	Subtotal		9.0	8.9	5.7	4.6	5.5	3.9
Terpenoid	(*Z*)-Citral	1685	-	-	0.2(0.03) ^a^	0.1(0.01) ^b^	-	0.1(0.01) ^b^
	(R)-(+) -β-Citronellol	1785	1.4(0.1) ^b^	2.2(0.2) ^a^	2.2(0.03) ^a^	1.5(0.1) ^b^	0.2(0.1) ^d^	0.6(0.2) ^c^
	Epoxylinalool	1750	-	-	-	1.8(0.4) ^b^	2.0(0.2) ^b^	3.0(0.6) ^a^
	Geraniol	1863	8.9(1.7) ^d^	14.6(0.5) ^c^	24.8(2.7) ^b^	29.7(2.0) ^a^	7.3(0.6) ^d^	10.0(0.8) ^d^
	Hotrienol	1632	2.1(0.1) ^bc^	1.9(0.1) ^bc^	2.4(0.3) ^ab^	2.6(0.04) ^a^	1.6(0.2) ^c^	2.6(0.3) ^a^
	D-Limonene	1204	0.1(0.01) ^c^	0.1(0.02) ^c^	0.1(0.01) ^c^	0.2(0.02) ^b^	0.2(0.04) ^b^	0.3(0.1) ^a^
	Linalool	1571	37.9(4.7) ^d^	37.1(3.3) ^d^	40.3(4.7) ^cd^	64.2(8.4) ^c^	90.4(3.6) ^b^	189.1(19.1) ^a^
	(*E*)-Linalool oxide	1451	-	-	-	0.04(0.002) ^b^	0.1(0.02) ^a^	0.1(0.01) ^a^
	β-Myrcene	1181	-	0.04(0.01) ^c^	0.1(0.003) ^b^	0.1(0.003) ^b^	0.1(0.01) ^b^	0.2(0.02) ^a^
	Nerol	1814	1.5(0.2) ^d^	3.5(0.6) ^c^	4.6(0.4) ^b^	6.3(0.7) ^a^	1.6(0.1) ^d^	2.1(0.3) ^d^
	Nerol oxide	1481	1.7(0.1) ^a^	1.8(0.1) ^a^	0.6(0.1) ^c^	1.2(0.2) ^b^	-	-
	(*E*)-β-Ocimene	1266	0.1(0.05) ^d^	0.2(0.03) ^cd^	0.2(0.02) ^c^	0.3(0.04) ^bc^	0.4(0.1) ^b^	0.6(0.02) ^a^
	(*Z*)-β-Ocimene	1251	0.2(0.03) ^e^	0.3(0.01) ^de^	0.3(0.01) ^cd^	0.2(0.01) ^bc^	0.3(0.04) ^b^	0.6(0.01) ^a^
	α-Phellandrene	1168	-	0.01(0.001) ^b^	0.01(0.003) ^b^	0.01(0.002) ^b^	0.01(0.002) ^b^	0.04(0.01) ^a^
	β-Phellandrene	1210	-	0.02(0.01) ^b^	0.02(0.001) ^b^	0.03(0.001) ^b^	0.03(0.01) ^ab^	0.05(0.01) ^a^
	β-Pinene	1177	0.2(0.01) ^e^	0.4(0.02) ^d^	0.5(0.01) ^c^	0.7(0.02) ^b^	0.6(0.03) ^b^	0.8(0.09) ^a^
	(*Z*)-Rose oxide	1358	0.01(0.002) ^e^	0.01(0.002) ^de^	0.02(0.002) ^c^	0.01(0.001) ^cd^	0.03(0.002) ^b^	0.04(0.001) ^a^
	Terpinen-4-ol	1606	-	-	-	-	0.04(0.01)	0.1(0.01)
	γ-Terpinene	1242	-	0.04(0.01) ^c^	0.1(0.01) ^a^	0.05(0.01) ^ab^	-	-
	α-Terpineol	1706	1.0(0.2) ^d^	10.8(0.2) ^c^	11.5(1.1) ^c^	16.4(0.2) ^b^	16.8(0.7) ^b^	26.2(3.9) ^a^
	Terpinolene	1278	-	0.04(0.02) ^c^	0.1(0.004) ^c^	0.1(0.01) ^b^	0.2(0.01) ^a^	0.2(0.04) ^a^
	Subtotal		55.1	73.1	88.1	125.6	121.9	236.7
	Total		2415	3106	3634	3802	4163	3082

^1^ RI: Kovat retention index. The concentrations of the free aroma compounds were expressed as Mean (SD) in ug/L. Different letters (a–e) within the same row indicate significant differences at *p* < 0.05.

**Table 3:** Odor thresholds, description, and activity values of volatile free aroma compounds.

Aroma Compounds	Odor Type	Odor Threshold (ug/L)	Odor Activity Value (OAV)					
			12 ^1^ WAFB	14 WAFB	16 WAFB	18 WAFB	20 WAFB	22 WAFB
2-Ethyl-1-hexanol	Citrus	270	0.015	0.016	0.017	0.02	0.02	0.017
1-Heptanol	Green	425	0.001	0.001	0.001	-	-	-
1-Hexanol	Herbal	500	0.337	0.734	1.449	1.877	1.308	0.806
(*E*)-2-Hexen-1-ol	Fruity	100	2.947	8.936	6.231	4.393	2.791	2.795
(*E*)-3-Hexen-1-ol	Green	1000	-	0.001	-	0.0003	0.001	0.001
(*Z*)-3-Hexen-1-ol	Green	70	0.473	0.613	0.771	0.644	0.416	0.247
1-Nonanol	Floral	50	0.004	0.002	0.002	0.002	0.002	0.002
(*E*)-2-Hepten-1-ol	Fatty	N.A	N.A	N.A	N.A	N.A	N.A	N.A
1-Octanol	Waxy	110	0.005	0.005	-	-	0.003	0.005
1-Octen-3-ol	Earthy	1	0.1	0.2	0.2	0.2	0.1	0.1
(*Z*)-2-Penten-1-ol	Green	720	-	0.001	0.0004	0.001	0.002	0.003
Benzaldehyde	Fruity	350	0.008	0.006	0.007	0.011	0.005	0.005
Hexanal	Green	4.5	171.911	173.733	237.6	245.444	365.244	314.378
(*E*)-2-Heptenal	Green	0.8	1	1	-	-	-	-
(*E*)-2-Hexenal	Green	17	62.765	54.288	62.041	66.3	83.029	41.776
(*Z*)-3-Hexenal	Green	0.25	17.2	15.2	18.4	20.8	34	26.4
Nonanal	Aldehydic	1	0.3	0.3	0.4	0.3	0.2	0.1
(*E*)-2-Octenal	Fatty	3	-	-	-	0.067	-	-
Methyl salicylate	Minty	40	0.018	0.035	0.01	0.008	0.001	0.001
Ethyl octanoate	Waxy	194	0.001	-	-	-	-	-
(*E*)-β-Damascenone	Fruity	0.002	340	105	75	10	10	20
2-Heptanone	Cheese	140	0.006	0.009	0.004	0.001	0.002	0.001
sulcatone		50	0.056	0.072	0.066	0.078	0.092	0.062
2-Octanone	Earthy	50	0.082	0.066	0.034	0.01	0.008	0.008
(*Z*)-Citral	Citrus	30	-	-	0.007	0.003	-	0.003
(R)-(+)-β-Citronellol	Floral	40	0.035	0.055	0.055	0.038	0.005	0.015
Epoxylinalool	Floral	6	-	-	-	0.3	0.333	0.5
Geraniol	Floral	40	0.223	0.365	0.62	0.743	0.183	0.25
Hotrienol	Tropical	110	0.019	0.017	0.022	0.024	0.015	0.024
D-Limonene	Citrus	10	0.01	0.01	0.01	0.02	0.02	0.03
Linalool	Floral	6	6.317	6.183	6.717	10.7	15.067	31.517
(*E*)-Linalool oxide	Floral	6	-	-	-	0.007	0.017	0.017
β-Myrcene	Spicy	36	-	0.001	0.003	0.003	0.003	0.006
Nerol	Floral	300	0.005	0.012	0.015	0.021	0.005	0.007
Nerol oxide	Green	3000	0.001	0.001	0.0002	0.0004	-	-
(*E*)-β-Ocimene	Floral	340	0.0003	0.001	0.001	0.001	0.001	0.002
(*Z*)-β-Ocimene	Floral	34	0.006	0.009	0.009	0.006	0.009	0.018
α-Phellandrene	Terpenic	8	-	0.001	0.001	0.001	0.001	0.005
β-Phellandrene	Minty	8	-	0.003	0.003	0.004	0.004	0.006
β-Pinene	Herbal	140	0.001	0.003	0.004	0.005	0.004	0.006
(*Z*)-Rose oxide	Floral	0.5	0.02	0.02	0.04	0.02	0.06	0.08
Terpinen-4-ol	Spicy	130	-	-	-	-	0.0003	0.001
γ-Terpinene	Terpenic	1000	-	0.00004	0.0001	0.00005	-	-
α-Terpineol	Terpenic	330	0.003	0.033	0.035	0.05	0.051	0.079
Terpinolene	Herbal	200	-	0.0002	0.001	0.001	0.001	0.001

^1^ WAFB: Weeks after full bloom; OAVs > 1 were presented in red; N.A: Not available.

**Table 4:** Changes in the sensory attribute scores of Shine Muscat grapes during ripening.

^1^ WAFB	Sweetness	Sourness	Astringency	Firmness	Flavor	Overall Acceptance
16	3.19 ± 0.8 ^b^	3.68 ± 0.8 ^a^	3.97 ± 1.1 ^a^	3.66 ± 1.1 ^a^	3.00 ± 1.0 ^b^	3.48 ± 1.0 ^b^
18	3.53 ± 1.2 ^ab^	3.76 ± 1.2 ^a^	3.83 ± 1.2 ^a^	3.63 ± 1.1 ^a^	2.97 ± 1.1 ^b^	3.39 ± 0.8 ^b^
20	4.26 ± 1.1 ^a^	3.76 ± 0.9 ^a^	4.00 ± 1.2 ^a^	3.52 ± 1.0 ^a^	4.13 ± 1.5 ^a^	4.20 ± 1.1 ^a^
22	4.23 ± 1.1 ^a^	3.59 ± 1.5 ^a^	3.96 ± 0.5 ^a^	3.43 ± 0.7 ^a^	4.04 ± 1.0 ^a^	4.16 ± 0.9 ^a^

^1^ WAFB: Weeks after full bloom. Different letters (a, b) within the same column indicate significant differences at *p* < 0.05.

## Data Availability

Data is contained within the article.
